# The Intradiscal Osteotomy: An Alternative Technique for Adult Spinal Deformity Correction

**DOI:** 10.7759/cureus.19062

**Published:** 2021-10-26

**Authors:** Wyatt L Ramey, Andrew S Jack, Rod J Oskouian, Robert A Hart, Jens Chapman

**Affiliations:** 1 Neurosurgery, Houston Methodist Neurological Institute, Houston, USA; 2 Neurosurgery, University of Alberta, Edmonton, CAN; 3 Neurosurgery, Swedish Neuroscience Institute, Seattle, USA

**Keywords:** interbody cage, spine revision surgery, degenerative spine disease, thoracolumbar spine, pedicle subtraction osteotomy, scoliosis surgery, adult spine deformity

## Abstract

Adult spinal deformity (ASD) correction has changed considerably since the initial description of a Smith-Petersen osteotomy (SPO), including pedicle subtraction osteotomies (PSO), and more minimally invasive techniques. Here, we introduce and describe the intradiscal osteotomy (IDO), a novel variation of Schwab type 3 and 4 osteotomies allowing pedicle and vertebral body preservation, and its advantages and disadvantages.

After pedicle screw placement, the posterior elements (except pedicles) are removed from the appropriate vertebrae, including the superior/inferior articulating processes, laminae, and spinous processes. An osteotome is used to remove the posterior aspect of the superior and inferior endplate, followed by the entire disc, creating more working room for eventual cage insertion. After the careful release of the annulus, an intradiscal distractor is used to distract the endplates and allow interbody cage insertion as anteriorly as possible. Pedicle and vertebral body preservation allow increased fixation and endplate cage support, which lengthens the anterior column and acts as a fulcrum when compressing posteriorly to restore lordosis. By allowing for anterior and posterior column release, the IDO technique provides a feasible, all-posterior approach for the correction of fixed or flexible kyphoscoliotic curves.

This technical report introduces and describes the IDO as an alternative method for thoracic and/or lumbar ASD correction. More studies are required to fully elucidate its outcome vs. complication profile compared to other deformity correction techniques.

## Introduction

The advent of modern spinal deformity surgery has been revolutionized by the development of spinopelvic parameters and the recognition of the importance of sagittal alignment [1]. These spinopelvic parameters have been shown to affect health-related quality of life (HRQOL) and reliably predict patient outcomes [2]. As such, one of the goals of adult spinal deformity (ASD) correction is to restore these spinopelvic parameters (for example, sagittal balance) for which multiple techniques have been established. Most notably, the three-column pedicle subtraction osteotomy (PSO) and extensions of it (Schwab grade 3 and 4 osteotomies) have become a mainstay for the treatment of thoracolumbar deformity due to its ability to shorten the posterior and middle spinal columns while hinging on the anterior column [3-5]. However, in comparison to contiguous Schwab grade 1 and 2 osteotomies, PSOs are a major undertaking with significant blood loss and are technically challenging [6]. Additionally, rates of pseudoarthrosis and rod fracture are exceptionally high due to biomechanical stress at or adjacent to the level of the PSO [7].

A three-column release in the presence of anterior column support is an ideal biomechanical solution for large, fixed thoracolumbar curves. In cases requiring open surgical correction, a three-column osteotomy can be performed while also providing anterior column support by performing an intradiscal osteotomy (IDO) and placement of an interbody cage. This acts to lengthen the anterior and middle columns before variably shortening the posterior column through compression in order to restore lordosis by the cage acting as a fulcrum around which the osteotomy can be reduced. In doing so, more lordosis can be achieved relative to multi-level grade 1 and 2 resections without the inevitable challenges and biomechanical stress of grade 3 and 4 resections due to pedicle and vertebral body preservation. The IDO provides an exclusively posterior approach to ASD correction while avoiding pitfalls associated with “spine-shortening” grade 2, 3, and 4 osteotomies. In this technical report, we aim to introduce and provide the first description of the intradiscal osteotomy, a variation of the Schwab grade 3 and 4 osteotomies, as an alternative means of restoring spinopelvic parameters and achieving adequate ASD correction.

## Technical report

The IDO can be readily performed in both the thoracic and lumbar spine. In order to gain access to the disc space and vertebral bodies, after pedicle screw placement, the posterior elements of the corresponding vertebrae must be removed with the exception of the pedicles (a demonstration of which can be found at www.ssftv.org; see Appendix). The superior and inferior articulating processes along with the laminae and spinous process are drilled or rongeured so they are flush with each pedicle above and below the disc space bilaterally. Care should be taken to avoid violating the pedicle during this part in order to prevent compromising this point of fixation’s strength and exposing the pedicle screw threads.

**Figure 1:**
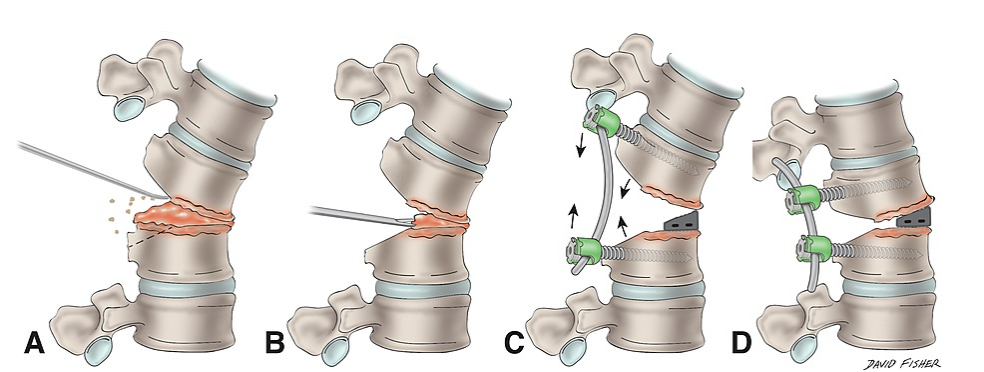
Schematic of the intradiscal osteotomy. (A) The posterior elements of the corresponding vertebrae including the spinous process, laminae, and superior/inferior articulating process are removed leaving only the pedicles. The posterior aspect of the endplates is also removed via osteotome. (B) Osteotomized bone fragments and intervertebral disc are removed. (C) and (D) After endplate preparation, the tallest possible interbody cage is inserted as anteriorly as possible in order to act as a fulcrum when compressing the pedicle screw-rod construct across the site of the osteotomy.

Once the disc space is adequately prepped on either side, a quarter-inch curved osteotome is used to perform the osteotomy while protecting the exiting nerve root. The posterior aspect of the superior and inferior endplate is removed along with the entire disc (Figure 1).

The removal of the posterior aspect of the endplates allows for increased working room for interbody device/cage and bone graft placement into the disc space, as well as facilitates compression posteriorly after cage placement to help restore sagittal balance (see below). However, care must be taken to avoid resecting too much of the endplates and violating them while they are being prepared with curettes in order to help prevent subsidence, and provide adequate surface area for placement of a cage and promote fusion.

Once the bone and disc material are removed with disc shavers, an intradiscal distractor is placed on one side of the disc space while on the opposite side progressively larger dilators and trials can be used to help finish releasing the annular attachments circumferentially. In the event of a fixed deformity (due to lateral osteophytosis, for example), a Cobb elevator and/or the sequential use of distractors and progressively larger shavers and dilators/trials can be used to gently break the osteophytes and distract the adjacent endplates in a controlled fashion. The now prepared interbody space is packed with fusion adjuncts such as bone graft followed by interbody cage placement. The cage is then positioned as anteriorly as possible in order to provide an anterior fulcrum for kyphosis correction upon posterior compression (Figures 1C, 1D). Unlike grade 3 and 4 osteotomies where the spinal column is shortened, the use of the distractors, annular release, and interbody cage(s) results in spinal column lengthening prior to a variable degree of posterior column shortening upon compression. While the operative techniques vary greatly from that of performing grade 3 and 4 osteotomies, the desired results are similar. The IDO is similar to a transforaminal lumbar interbody fusion (TLIF) in that the facet is obliterated in order to place an interbody cage; however, the bony work within the confines of the posterior disc space is more extensive and deliberate in order to achieve sagittal correction when compressing posteriorly, which is not always performed with a standard TLIF.

**Figure 2:**
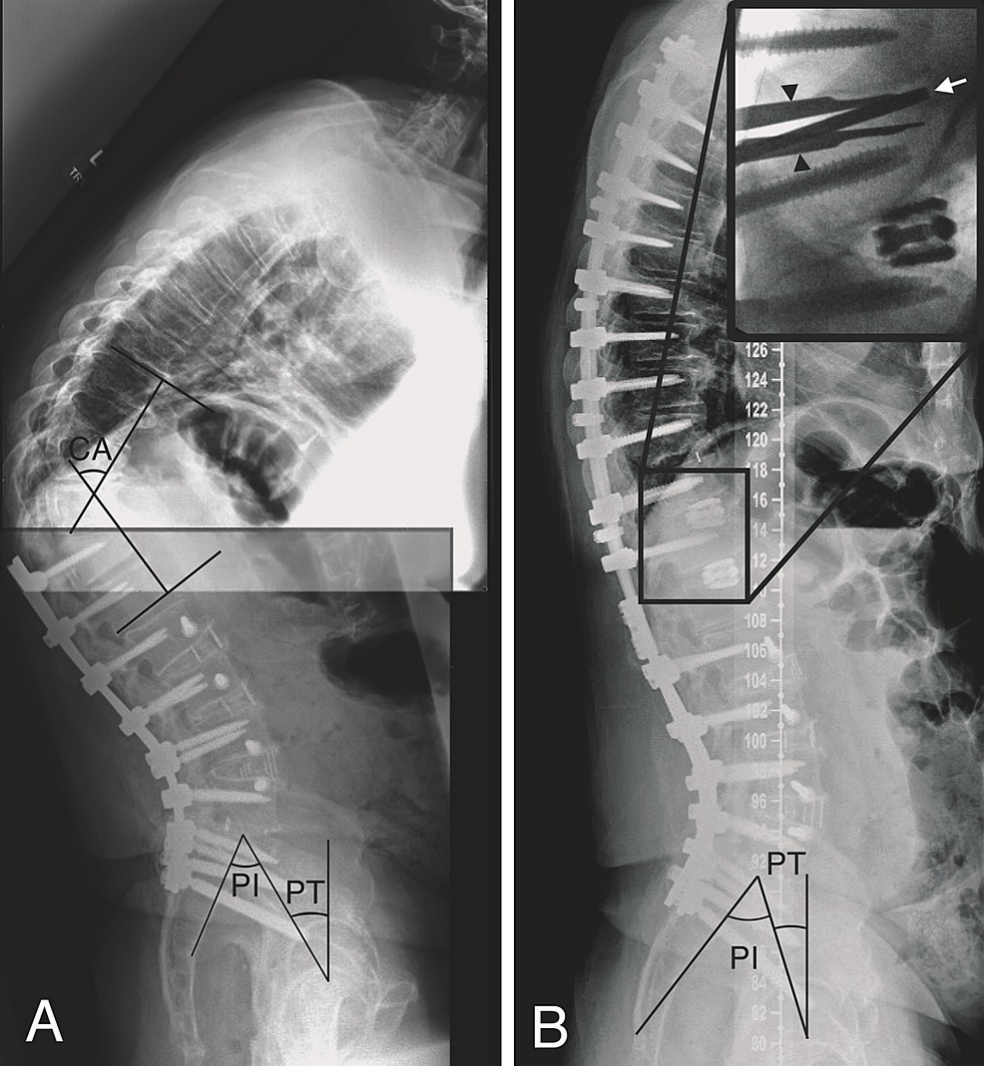
Pre- and postoperative radiographs of the intradiscal osteotomy. (A) Preoperative lateral radiograph showing a proximal junctional failure (PJF) after previous T12-pelvis instrumented fusion. Cobb angle (CA) = 69.7 degrees, pelvic incidence (PI) = 53.7 degrees, and pelvic tilt (PT) = 30.5 degrees. (B) Postoperative lateral radiograph after PJF correction using IDOs at the T12-L1 and T11-T12 segments (shown via intraoperative fluoroscopy in inset) resulting in CA and PT correction. Black arrowheads: disc space distractor; white arrow: disc space shaver. IDO, intradiscal osteotomy.

Several different types of interbody cages can be used. In the lumbar spine and low thoracic spine, bilateral titanium expandable lordotic cages can be used with relative ease to further augment correction below the spinal cord (Figure 2).

In the upper and mid-thoracic spine, bilateral titanium straight cages or a curved polyetheretherketone (PEEK) cage are typically employed with again emphasis on placing them anteriorly. When inserting the interbody cage(s), it is important that an assistant holds the intradiscal distractor (maintain distraction) as the reverberation from mallet use can cause the distractor to release. Furthermore, when placing bilateral expandable cages (Figure 2), it is important to place the cages anteriorly and laterally close to the apophyseal ring where the endplate bone is strongest (to avoid subsidence, as well as the cages colliding in the middle of the disc space). Moreover, with bilateral expandable cages, it is important to expand the cages simultaneously (and not consecutively) in order for each cage to share the force of distraction and avoid having one “torque-out” and becoming loose within the disc space.

Once each cage is placed and the pedicle screws are properly positioned, the rod is used to compress across the levels where IDOs were performed. In this case, the spinal column has been first lengthened, and then the posterior column is subsequently shortened to a variable extent through compression. The cages act as an anterior column fulcrum in order to provide lordosis and sagittal plane correction. In addition to there being increased working room for interbody cage placement, removal of the posterior aspects of the superior/inferior endplates through osteotomy play another important role during this stage. During posterior compression, the IDO prevents the posterior aspects of the vertebral bodies from “binding” against one another, which would then move the axis of rotation during the sagittal correction to the posterior disc space and potentially result in the anterior interbody cage loosening by distracting the anterior column. Finally, in the event of a coronal plane deformity, an interbody cage may be placed asymmetrically with contralateral compression to facilitate the correction.

## Discussion

The intradiscal osteotomy provides a powerful and efficient means of correcting ASD while providing durable anterior column support. Modern techniques for major spine deformity correction consist primarily of posterior osteotomies (Schwab osteotomy grades 2-4), in addition to staged minimally invasive surgery (MIS) procedures. In this technical report, we sought to introduce and detail the IDO as an alternative variation of the more common deformity correction techniques.

IDO advantages

Several advantages exist with the IDO technique over the Schwab grade 3 and 4 osteotomies. Unlike the former, the IDO does not require pedicle or vertebral body resection. This allows for added pedicle screw fixation at the site of deformity correction, which could potentially reduce the incidence of pseudarthrosis near the osteotomy, but this would need to be confirmed with further studies. Furthermore, maintaining pedicle and vertebral body integrity also potentially increases the segmental stability of the pedicle screw-rod and interbody device construct resulting in it seeing less stress. Packing the interbody space with bone graft and/or bone fusion adjuncts can also help promote fusion and help mitigate the risk of pseudoarthrosis. Preserving the pedicle and vertebral body also helps to decrease both the length of the procedure and estimated blood loss (EBL). However, the amount of segmental correction achieved via the IDO in our experience (15-20°) is less than what has been described for more traditional Schwab grade 3, 4, and 5 (PSOs and vertebral column resections) corrections (30° and 30-60°, respectively), and more than Schwab grade 1 and 2 osteotomies (10° per level) [3,6,8,9]. Finally, with expandable cages and using the IDO, a surgeon can tailor the amount of sagittal and coronal deformity correction at any particular level, while taking advantage of anterior column reconstruction, release, and fusion through a single-stage, posterior-only approach that almost every spinal surgeon is familiar with.

IDO disadvantages

Although there are many advantages to our described techniques outlined above, there are also several drawbacks. One of the most salient drawbacks relates to surgical cost. With the use of bilateral expandable interbody cages at several levels, the surgical cost escalates rapidly. Many of the other disadvantages to performing this technique are those similarly affecting Schwab grade 1 and 2 osteotomies. However, one that is perhaps unique to the IDO is the delayed presentation of patients with a sacral insufficiency fracture. We prefer to use expandable interbody devices in order to maximize our sagittal deformity correction. Since beginning this technique, two patients have presented in a delayed fashion with an insufficiency fracture at the S1 endplate, even with pelvic fixation, potentially related to the use of expandable vs. static interbody cages. Furthermore, the IDO also does not achieve the same degree of sagittal correction as other techniques such as Schwab grade 3-5 osteotomies (though it does provide more than Schwab grade 2). In the case of an acute, fixed, kyphotic deformity, an IDO is likely not going to achieve the maximum amount of correction over a short segment (as would be seen with PSO or vertebral column resection [VCR]). In addition to this (and more like Schwab grade 2/Smith-Petersen osteotomy [SPO]), the more levels at which an IDO is performed, the longer the procedure and the higher the EBL.

## Conclusions

The intradiscal osteotomy is an effective means of correcting thoracolumbar deformity while also providing anterior column support. While other techniques of deformity correction remain viable and effective options, IDO is an additional technical variation that may reduce the overall invasiveness associated with the PSO. The IDO is a technique that has yet to be extensively studied in the realm of ASD surgery and ongoing studies are needed to assess its indications, complication rates, and effectiveness.

## Appendices

Video demonstration of the intradiscal osteotomy

https://www.youtube.com/watch?v=RsYHlyZ4sPs&list=PLyptnEaqO5i6CC1nMm1zX5KzIsk_SC_Ex&index=5
